# Natural Products as Promising Drug Candidates for the Treatment of Alzheimer’s Disease: Molecular Mechanism Aspect

**DOI:** 10.2174/1570159X11311040005

**Published:** 2013-07

**Authors:** Niloufar Ansari, Fariba Khodagholi

**Affiliations:** Neuroscience Research Center, Shahid Beheshti University of Medical Sciences, Tehran, Iran

**Keywords:** Alzheimer’s disease, Amyloid β, Apoptosis, Natural products, Neuroprotection, Tau protein.

## Abstract

Alzheimer’s disease (AD) is the most common neurodegenerative disorder to date, with no curative or preventive therapy. Histopathological hallmarks of AD include deposition of β-amyloid plaques and formation of neurofibrillary tangles. Extent studies on pathology of the disease have made important discoveries regarding mechanism of disease and potential therapeutic targets. Many cellular changes including oxidative stress, disruption of Ca2+ homeostasis, inflammation, metabolic disturbances, and accumulation of unfolded/misfolded proteins can lead to programmed cell death in AD. Despite intensive research, only five approved drugs are available for the management of AD. Hence, there is a need to look at alternative therapies. Use of natural products and culinary herbs in medicine has gained popularity in recent years. Several natural substances with neuroprotective effects have been widely studied. Most of these compounds have remarkable antioxidant properties and act mainly by scavenging free radical species. Some of them increase cell survival and improve cognition by directly affecting amyloidogenesis and programmed cell death pathways. Further studies on these natural products and their mechanism of action, parallel with the use of novel pharmaceutical drug design and delivery techniques, enable us to offer an addition to conventional medicine. This review discussed some natural products with potential neuroprotective properties against Aβ with respect to their mechanism of action.

## 1. INTRODUCTION

Alzheimer’s disease (AD) is a progressive neuro-degenerative disorder that is characterized by the loss of memory and cognitive impairments [1]. Many biochemical changes within cell have been identified to induce neuronal cell death. Oxidative stress, disruption of Ca^2+^ homeostasis, inflammation, metabolic disturbances, and accumulation of unfolded/mis-folded proteins are among cellular changes that finally lead to programmed cell death in AD [2]. 

Histopathological hallmarks of AD include deposition of β-amyloid (Aβ) plaques and formation of neurofibrillary tangles [3]. Indeed, AD is a protein mis-folding disease, called “proteopathy”. Senile plaques are made of insoluble Aβ peptides that are formed by proteolytic fragmentation of amyloid precursor protein (APP), a trans-membrane protein that penetrates through the neuronal membranes [4]. Neurofibrillary tangles are made of aggregates of hyperphosphorylated tau, a microtubule-associated protein [5].

Most of the researches in AD during past two decades have focused on “amyloid hypothesis”. This hypothesis postulates that Aβ deposition is the initial event in neuronal dysfunction in AD. The support given for this hypothesis is the dementia of patient with Down’s syndrome (trisomy 21). As APP genes locate in chromosome 21, it has been proposed that their mutations result in cognitive impairment and dementia [6].

APP cleavage by β-secretase at extracellular domain, and by γ-secretase at the trans-membrane region, leads to formation of Aβ proteins [7]. Gamma-secretase complex consists of at least four proteins, including Presenilin (PS). PS, an aspartyl protease, is the catalytic subunit of the enzyme and its mutation causes alterations in APP processing and increases the amount of toxic Aβ [8]. Although amyloid plaques are the major characteristic of AD pathology, their presence does not necessarily mean the increased amyloid production. Decrease of clearance could also lead to this accumulation. It has been also reported that some soluble species of Aβ are more toxic than Aβ aggregates [9]. Thus, the main focus has been made on preventing the formation of Aβ from its precursor. 

Although several compounds have been proposed to decrease neurodegeneration in AD models, limited approved drugs are available for the management of AD patients. A major part of today’s pharmaceutical market is the use of natural products. The use of drug substances derived from natural sources has a long tradition in medicine [10, 11].

This review article describes the mechanism of AD, current medications for AD and existed problems in developing new medications. We also summarize the potential of some natural products for inhibiting Aβ-induced cell death and recent findings on their mechanisms of action. Although Aβ is not the only factor capable of causing cognitive decline in AD, due to extent of compounds and experimental models, in this paper we focused on the active substances reported to inhibit Aβ-induced cognitive impairment or cell death.

## 2. MECHANISM OF NEURONAL CELL DEATH IN AD

Oxidative stress is the imbalance between prooxidants and antioxidant factors that lead to accumulation of reactive oxygen species (ROS) [12]. This reactive species can lead to cell membrane lipid destruction, DNA cleavage, oxidation of proteins, and finally apoptosis [13]. Apoptosis is the predominant type of neuronal cell loss observed in AD [14]. Apoptotic cell death signaling can be divided into two major pathways; intrinsic (or mitochondrial) pathway and extrinsic (or death receptor- mediated) pathways [15].

**Fig. (1):**
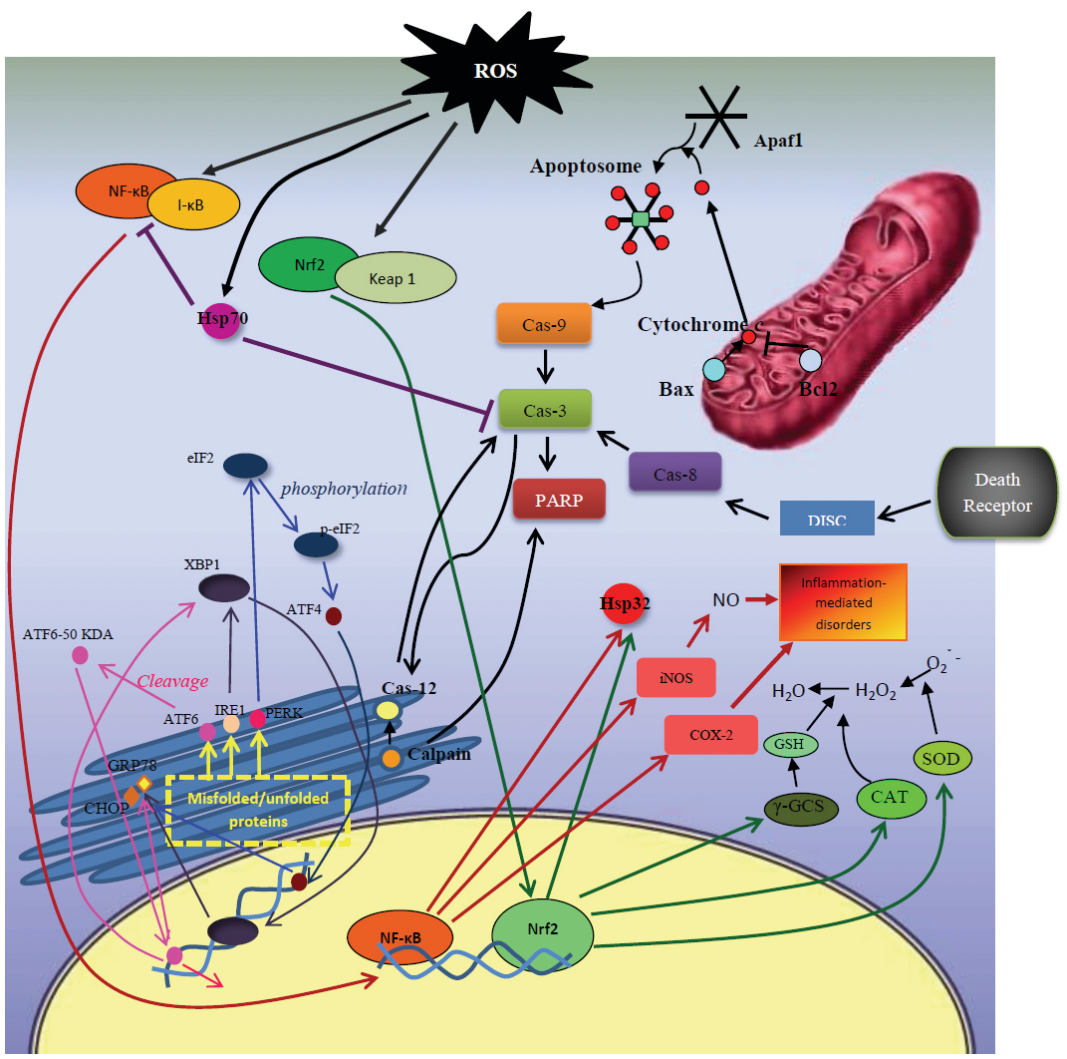
Mechanism of apoptotic pathways and ER stress.

Apoptosis in mammalian cells regulates by a large number of proteins (Fig. **1**). In the intrinsic pathway, stimuli acts directly or indirectly on mitochondria and affects mainly Bcl-2 family and caspase [16].

Bcl2 superfamily consists of both pro-apoptotic (such as Bax, Bad, and Bak) and anti-apoptotic (such as Bcl-2 and Bcl-xL) proteins. Decrease of anti-apoptotic protein and/or increase of pro-apoptotic factors results in disruption of mitochondria membrane potential, swelling of mitochondrial membrane, and release of cytochrome c to cytoplasm [17]. In cytoplasm, cytochrome c forms a multi-molecular holoenzyme complex with apoptotic protease activating factor 1 (Apaf1), which cleaves procaspase-9 to its active form. Active caspase-9 then cleaves procaspase-3 and initiates the caspase cascades [18].

Extrinsic pathway of apoptosis involves the interaction of death signals, for example tumor necrosis factor (TNF-α) with death receptors, such as tumor necrosis factor receptors 1 (TNFR1), and formation of death-inducible signaling complex that activates caspase-8, which could also cleave procaspase-3 to its active form [19].

Activated caspase-3, in both intrinsic and extrinsic pathways, activates poly (ADP-ribose) polymerase (PARP) and other death substrates, such as APP, PS1 and PS2 proteins [20].

Stress conditions also affect the folding of proteins in endoplasmic reticulum (ER) lumen [21]. Three main ER pathways involved in folding include inositol-requiring enzyme 1 (IRE1), protein kinase RNA-like ER kinase (PERK), and activating transcription factor 6 (ATF6) [22]. ER stress response is mediated *via *three different signaling pathways: unfold protein response (UPR), which increases the level of chaperones; ER-associated protein degradation (ERAD) that degrades the misfolded proteins by activating ubiquitin/proteosomal pathway; and ER overload response (EOR) which is induced when ER is overload with proteins that are not transported to Golgi complex [23]. Under ER stress conditions, glucose-regulated protein 78 (GRP78) which is an ER chaperone, dissociates from ATF6, PERK, and IRE1 and binds to malfolded proteins to facilitate their folding [24]. C/EBP homologous protein (CHOP) together with caspase-12, which are ER resident caspases, and calpain mediate ER stress-induced apoptosis by affecting executioner caspases, such as caspase-3 [25,26].

While accumulation of unfolded proteins in ER provokes these pathways, accumulation of misfolded proteins in the cytosol leads to increased expression of heat shock proteins (HSPs) which act as molecular chaperons [27]. HSPs expression is induced by several stimuli including heat shock, ischemia damage, infection, and heavy metals [28]. HSPs may protect cells by mechanisms unrelated to their chaperone function through inhibition of apoptosis [29]. Stress-inducible Hsp70 is a prominent cytoprotective factor that protects the sensitive sites of the target proteins and thereby acts as a cytoplasmic “antioxidant” [30].

In addition to mitochondria- and ER-resident proteins, many stress-sensing transcription factors are also activated in AD.

NF-E2 related factor 2 (Nrf2) is a central transcription factor involved in transcriptional activation of phase II detoxifying enzymes *via *antioxidant response element (ARE) [31]. Release of Nrf2 from its cytoplasmic inhibitor, Kelch-like ECH-associated protein 1 (Keap1), leads to activation of Nrf2 and its translocation to nucleus, where it activates transcription of ARE-driven genes, such as Hsp32 and γ-glutamylcysteine synthetase (γ-GCS) [32].

Nuclear factor-κB (NF-κB) is another transcription factor that is activated by TNF-α, interleukin 1β (IL-1β) and lipopolysaccharide (LPS) (canonical pathway) or by LTα/β, CD40 ligand (non-canonical pathway) [33]. In unstimulated cells, NF-κB is sequestered inactive in cytoplasm by binding to IκBs (Inhibitor of κB). Activation of the NF-κB involves the phosphorylation of two serine residues located on IκB regulatory domain by IκB kinase (IKK) and release of NF-κB [34]. In nucleus, NF-κB induces production of different mediators, like nitric oxide (NO), and regulates a number of inflammation- and oxidative stress-related genes, such as cyclooxygenase 2 (COX-2), superoxide dismutase (SOD), glutamate receptors, growth factors (brain-derived neurotrophic factor (BDNF) and nerve growth factor(NGF)), and cytokines (TNF-α and TNFR) [35,36]. NF-κB signaling is inhibited by peroxisome proliferator-activated receptor γ (PPARγ), a transcription factor of the nuclear hormone receptor superfamily [37]. PPARγ agonists attenuate effectively oxidative stress, inflammation and apoptosis in the central nervous system [38].

Mitogen-activated protein kinase (MAPK) cascades are other major signaling pathways involved in cell proliferation, differentiation and adaptation [39]. The p38 MAPK signaling has been widely accepted as a cascade contributing to neuroinflammation, excitotoxicity, synaptic plasticity and tau phosphorylation [40]. Inhibitors of ERK and p38 MAPK improve spatial learning and memory impairment in Aβ-injected rats by increasing phosphorylated cAMP-response element binding protein (CREB) level [41]. JNK protein is a stress activated protein kinase with several targets including Bcl family members and microtubule associated proteins, such as tau [42].

Another important signaling pathway involved in AD is Wnt pathway. Wnt signaling plays an important role in normal neural development and maintenance of neuronal homeostasis, synaptic plasticity, axonogenesis and establishment of brain polarity [43]. Activation of the Wnt pathway attenuates cytosolic glycogen synthase kinase 3β (GSK-3β) activity *via *protein kinase C (PKC) enzyme, thereby prevents phosphorylation and degradation of β-catenin and increases its nuclear translocation [44]. In nucleus, β-catenin interacts with TCF/LEF family trans-cription factors to promote specific gene expression [45]. These gene products are important in determining cell’s fate during normal development and in maintaining homeostasis [46]. Several studies have shown that PS-1 protein could form high molecular weight complexes with GSK-3β and β-catenin protein [47]. It has been suggested that PS-1 inherited mutations may affect the levels, trafficking or the phosphorylation state of cytosolic β-catenin [48].

## 3. CURRENT TREATMENTS

**Fig. (2):**
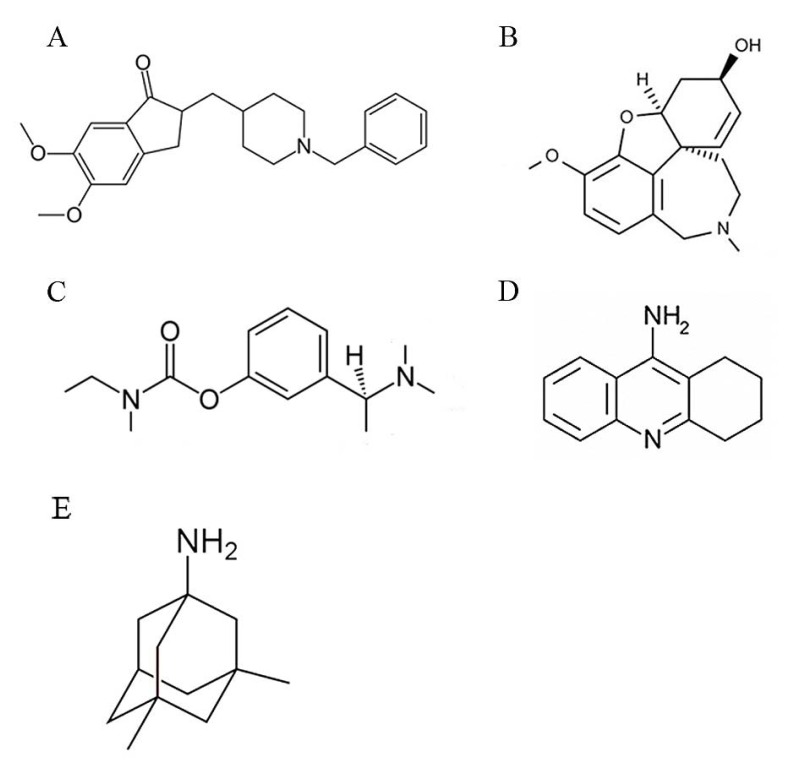
Chemical structure of FDA-approved drugs for AD. A) Donepezil; B) Galantamine; C) Rivastigmine; D) Tacrine; E) Memantine.

To date, there is no curative or preventive therapy for AD. Five medications have FDA approve for management of AD, all of them offer symptomatic benefits; tacrine, rivastagmine, donepezil, and galantamine are acetyl cholinesterase inhibitor (AChEI), while memantine is an N-methyl-D-aspartate (NMDA) receptor antagonist [49] (Fig. **2**).

It has been found that AD is associated with reduction of cholinergic neurons activity [50]. AChEIs reduce the rate of acetylcholine degradation, and thereby increase its concentration in the brain. All of these drugs are used in mild to moderate AD [51]. Donepezil is approved for the treatment of advanced stages of AD [52]. Memantine is also used in moderate to severe AD. This NMDA receptor antagonist blocks overstimulation of receptor by glutamate that could lead to excitotoxicity in AD [53].

As mentioned above, none of these drugs could delay the onset of AD or halting its progression [54]. Several problems exist in development of new therapeutics. First of all, there are mixed causes of dementia and neuropathology in many patients, particularly those who are older than 80 years [55]. Besides, in patients with different stages of the disease, it might be too early or too advanced for a disease-modifying effect of specific drugs [56]. Another problem in development of AD therapy is that several compounds with positive results in preclinical studies fail at clinical trials because of their low penetration across blood brain barrier (BBB), which limits their targeting. In recent years, several compounds have been reported for their neuroprotective effects in cellular and/or animal models of AD [57,58]. Although many of them were failed in different phases of clinical trials, some of them are promising candidates for AD treatment [59,60].

## 4. NATURAL PRODUCTS

Medicinal plants are important source of protective compounds against AD. Using the structure of these active substances as templates for synthetic drugs provides a wide range of potential neuroprotective compounds [61]. In the past few decades, several researches attempted to assess the effect of total plant extract on AD and to isolate the active substance responsible for the protective effects [62,63]. In this section, we review the most studied neuroprotective natural substances with focus on their mechanism of action (Table **1**).

### 4.1. Bilobalide

Bilobalide (BB) is the main terpenoid of *Ginkgo biloba* leaves with potent protective effects on neurons and Schwann cells [64]. This sesquiterpene trilactone induces liver enzymes CYP3A1 and 1A2, which may be partially responsible for interactions between Ginkgo and other herbal medicines or pharmaceutical drugs [65]. 

In rats, oral administration of Ginkgo extracts and/or pure BB caused dose dependent increase of BB plasma levels [66]. The plasma half-life of BB in human is about 2-3 h [67].

BB (25-100 µM) blocked ROS-induced apoptosis in early stages and decreased the elevated levels of p53, Bax and caspase-3 in PC12 cells [68]. BB (50 µl, 0.14 g/ml) also affected mitochondrial function by upregulating cytochrome c oxidase subunit I [69]. 

BB (10 µM) inhibited the β-secretase activity of cathepsin B and reduced generation of two β-secretase cleavage products of APP, Aβ and soluble APPβ, *via *PI3K-dependent pathway [70]. Additionally, GSK 3β signaling might be involved in BB (10 µM)-induced Aβ reduction as a downstream target of the activated PI3K pathway [71]. In hippocampal neurons, BB stimulated neurogenesis and synaptogenesis by increasing the levels of pCREB and BDNF [72]. Chen *et al*. reported that the capacity of BB to potentiate neurite outgrowth in developing nerves can be suppressed as its concentration is boosted to 400 µM. They suggested that an excessive dosage of BB could provoke adverse responses to the recovery of regenerated nerves [73].

### 4.2. Quercetin

Quercetin (QCT) is a polyphenolic flavonoid found in a wide variety of foods including capers, apples, onions, berries, green and black tea, and red wine [74]. This flavonol-type flavonoid acts as a bioactive compound, mainly by scavenging ROS and showing antioxidant properties [75]. In addition, it exerts several pharmacological effects, such as anti-cancer, antiviral, anti-inflammatory, and anti-amyloidogenic activities [76, 77]. In human studies, QCT (doses up to 1,000 mg/day) had no adverse effects on blood parameters of liver and kidney function, hematology, or serum electrolytes [74].

One characteristic feature of QCT is that the elimination of its metabolites from plasma is quite slow, with reported half-lives ranging from 11 to 28 h, which makes QCT accumulation in body in daily uptake [78]. Recently, solid lipid nanoparticles (SLNs) of QCT were prepared in order to improve its penetration *via *BBB. Behavioral studies confirmed that SLN-encapsulated QCT shows a better neuroprotective effect [79]. Most animal studies reported no toxicity/carcinogenicity, which may be due to extensive QCT metabolism by the intestinal and the liver cells. Liposomal preparations or higher BBB permeability conditions, by increasing the amount of QCT aglycone reaching the CNS parenchyma, may elevate the risk of neurotoxicity. Thus, further toxicological studies are needed to investigate risk/beneficial effects of liposomal preparations [80].

QCT (10 µM) showed anti-amyloidogenic effects by inhibiting the formation of Aβ fibrils [81]. Lower doses of QCT (5-20 µM) significantly attenuated Aβ-induced apoptosis in hippocampal cultures; however it induced cytotoxicity at high doses (40 µM) [82]. QCT combination with BB could significantly enhance phosphorylation of CREB and the level of BDNF in mice brain [83].

### 4.3. (-)-Epigallocatechin-3-gallate

(-)-Epigallocatechin-3-gallate (EGCG) is the most abundant catechin in green tea leaves (*Camellia sinensis*). Carob flour, a cocoa-like substance derived from the ground pods of the carob plant or *Ceratonia siliqua* has also a high content of EGCG. Apples, blackberries, strawberries, nuts, peaches, avocados, plums, onions and raspberries have lower amounts of EGCG [84]. During fermentation, many catechins are oxidized to theaflavin and thearubigen which provide darker colors of the black tea [85]. EGCG is a potent antioxidant flavonoid and has been the subject of many studies in cancer, atherosclerosis, and neurodegenerative diseases, such as AD [86].

Absorption of EGCG from the small intestine is largely by passive diffusion; however, at high concentrations, the small intestinal and colonic tissues become saturated [87]. After oral absorption, tea catechins undergo extensive methylation, glucuronidation, and sulfation. The elimination half-life of EGCG is about 3 h [88]. It has been shown that EGCG nanolipids oral bioavailability was two folds more than free EGCG and they show a better α-secretase enhancing effect [89].

Orally administered EGCG (10 mg/kg) could reduce AChE activity, glutathione peroxidase activity, NO metabolites and ROS content in streptozotocin-model of dementia [90]. In mutant PS2 AD mice, EGCG (3 mg/kg in drinking water) enhanced memory formation and α-secretase activity, and suppressed γ-secretase activity [91]. Rezai-zadeh *et al*. (2008) showed that EGCG administration, i.p. (20 mg/kg) and p.o. (50 mg/kg in drinking water), modulated tau profile and provided cognitive benefits to Tg mice [92]. Besides, EGCG (5-10 µM) exhibited potent iron-chelating activities and decreased both immature and full length cellular holo-APP [93]. 

EGCG (1.5 and 3 mg/kg in drinking water) also prevented LPS-induced memory impairment and apoptosis *via *non-amyloidogenic proteolysis by decreasing APP expression, beta-site APP cleaving enzyme 1 (BACE-1) activity, and Aβ levels. In this model of AD, EGCG (1, 10, and 100 µM) also prevented astrocytes activation and inflammatory factors including TNF-α, IL-1β, macrophage colony-stimulating factor, soluble intracellular adhesion molecule-1, IL-6, inducible nitric oxide synthase (iNOS) and COX-2 [94,95]. Pae *et al*. reported that dietary supplementation with high dose of EGCG (1 % w/w) promotes adverse response by inducing TNF-α, IL-6, and IL-1β and lipid inflammatory mediator PGE2 in mice splenocytes and macrophages [96].

EGCG (2 and 20 µM) also inhibited vascular endothelial growth factor, prostaglandin E2, p38, JNK, and MAPK phosphatase-1 [97]. Higher concentrations of EGCG (200 µM) induced cellular toxicity in human astrocytoma, U373MG cells [97].

EGCG (3 mg/kg in drinking water) inhibited apoptosis in Aβ1-42- injected mice brain by inhibiting ERK and NF-κB [91]. EGCG (10 µM) could prevent Aβ-induced impairment of NMDA, calcium influx, ROS production, and mitochondrial dysfunction in primary cortical neurons [98]. *In vitro* and *in vivo* studies done by Dragicevic *et al*. (2011) showed that EGCG and luteolin (Lu) were the top two mitochondrial restoration compounds among 25 tested flavonoids [99].

In addition to mentioned effects, EGCG could be also used as an enhanced supplement for Huperzine A (HupA). EGCG (10-300 mg/kg) enhanced the inhibitory effect of HupA on AChE by increasing its affinity for serum albumin. Upon addition of EGCG to HupA, a remarkably enhanced and prolonged inhibitory effect was detected [100].

### 4.4. Resveratrol

Resveratrol (RSV) belongs to a class of polyphenolic compounds called Stilbenes [101]. Stilbenes are produced by several plants after exposure to stress, injury, fungal infection, or UV radiation [102]. RSV is one of the main flavonoids of red wine and can be found in the skin of grapes and other fruits and nuts [103]. Several studies reported anti-cancer, anti-inflammatory, cardiovascular benefits, lowering blood glucose, and neuroprotective effects of RSV [104,105]. In short term study of repeated dose of RSV, no severe adverse effect of RSV was reported. Only 12.5% of the participants experienced frontal headache [106, 107].

RSV is well-absorbed from gastrointestinal lumen but it has low bioavailability due to its rapid metabolism and elimination [108]. RSV loaded lipid core nanocapsules increased RSV concentration in brain tissue, compared to free RSV [109].

RSV (10 and 20 mg/kg, p.o.) acts mainly as a potent antioxidant by scavenging ROS, increasing GSH level and ameliorating antioxidant capacity of the cell [110]. RSV (10 µM) also increases intracellular Ca^2+^ in cortical neurons *via *modulating secondary messengers, cGMP, cAMP, and NO. This enhancement of Ca^2+^ promotes cellular glucose utilization by inducing calcium dependent AMP-activated protein kinase [111]. 

RSV decreased the level of Aβ by inducing non-amyloidogenic cleavage of APP and increasing the clearance of Aβ [112]. Indeed, RSV (50 µM) bound to Aβ in different states; its binding response to fibrillar Aβ1-42 was higher than its monomer, but it bound to monomeric Aβ1-40 stronger than its fibrillar form [113]. Its high affinity to Aβ (at the concentration of 10^-4^ M in AD human brain) led researchers to design a new method of amyloid detection [113]. RSV specifically stained Aβ plaques and can be used as a reliable probe for amyloids. RSV (15, 45, and 135 mg/kg) can also inhibit AChE activity within cells [114].

RSV (2.5-40 µg/ml) inhibited LPS-induced inflammatory response by decreasing inflammatory factors, such as NO, TNF-α, IL-1β, and IL-6 in astrocytes [115]. RSV (100 and 200 µM) can also inhibit CRP protein and ERK1/2 MAPK [116]. Its inhibitory effect on LPS-induced NF-κB activation (50 µM) is mainly mediated by inhibiting IKK and IκB phosphorylation. NF-κB suppression resulted in decrease of downstreams levels, TNF-α and IL-6 [117, 118].

Combinational use of melatonin (1, 10, 50, 100, and 500 μM) with RSV (0.1, 1, 5, 10, and 20 μM) potentiated RSV effects by increasing Hsp32, reducing ROS level, restoration of mitochondrial membrane potential (MMP), increasing GSH, and phosphorylation of AMPK [119]. Co-treatment with low concentrations of melatonin (1-10 µM) and RSV (0.1-1 μM) that did not prevent Aβ1-42-induced cytotoxicity alone, synergistically protected HT22 hippocampal neuronal cells against Aβ1-42-induced toxicity [119].

### 4.5. Curcumin

Curcumin (CUR) is the principal curcuminoid of turmeric, popular Indian spice derived from the rhizomes of *Curcuma longa*, which is a member of the ginger family [120]. Curcuminoids are polyphenolic compounds that give turmeric its yellow color and can be used as a food coloring [121].

Many preclinical studies suggest that CUR may be useful for the prevention and/or treatment of several diseases, such as colorectal cancer, cystic fibrosis, inflammatory diseases and AD [122]. In phase I clinical studies, CUR (up to 8,000 mg/day, p.o.) did not result in significant side effects except mild nausea and diarrhea [123]. However, excess use of CUR can damage the gut microbiota, interfering with the normal physiology and immune response [124]. Bioavailability of orally administered CUR is relatively low and mostly metabolites of CUR, instead of CUR itself, are detected in plasma following oral consumption [125]. Its conjugation with aminoacids (such as isoleucine, phenylalanine, and valine) increases its α-secretase activation [126]. In addition, several drug delivery systems have been studied for better targeting of CUR, such as liposomes, polymeric nanoparticles, SLNs, micelles, nanogels, and complexes with dendrimer/dimer [127-132]. SLN CUR showed a great recovery in membrane lipids, as well as AChE activity, in AlCl_3_-treated mice and the results were comparable with those achieved by rivastigmine [133]. CUR (10 µM) also inhibited aluminum-induced oxidative stress and mitochondrial dysfunction in rat brain [134]. It can also directly bind to metals, especially iron and cupper, and thereby acts as a chelator [135].

CUR (5-10 µM) protected PC12 cells against Aβ-induced neurotoxicity by inhibiting oxidative damage, intracellular calcium influx, and tau hyperphosphorylation. Higher concentrations of CUR (50 µM) induced cellular toxicity in PC12 cells [136]. This polyphenol inhibited Aβ-induced mitochondrial depolarization of membrane potential and suppressed apoptotic factors cytochrome c, Bax and caspase-3. CUR (1-10 µM) also inhibited activated cyclin D1 protein level in Aβ-treated neurons [137]. It can also modulate cellular antioxidant enzymes, SOD and CAT. Both total and phosphorylated GSK-3β decreased in CUR-treated cells [138]. Inhibiting GSK-3β activated Wnt/β-catenin signaling and induced β-catenin (5-20 µM of CUR) [139]. CUR (5-20 µM) also inhibited GSK-3β-mediated PS1 activation and thereby decreased Aβ production [140]. Another study reported that CUR (10^-4^-10^-2^ %) promoted Aβ fibril conversion by reducing the pre-fibrillar/ oligomeric form of Aβ, resulting in reduction of neurotoxicity in Drosophila [141]. Mustuga *et al*. (2001) examined the binding of CUR (0.009 %) to senile plaques in brain species of several aged animals and human AD patients. Interestingly, they found that CUR specifically bound to aggregated amyloids in various animals, and further to phosphorylated tau protein, probably according to its conformational nature [142]. This binding affinity led to design of a bivalent ligand of CUR and cholesterol, BMAOI 14 that can rapidly cross BBB and stains monomeric, oligomeric and fibrillar Aβ [143,144]. Having many properties required for optical imaging and good BBB penetration made BMAOI 14 a good candidate of Aβ-imaging agent. Another group designed 1-(4-[^18^F]fluoroethyl)-7-(4’-methyl)curcumin as a PET radioligand for Aβ plaque imaging [144].

CUR (1 µM) ameliorated cognitive deficit by stimulating BDNF and increasing GSH levels in hippocampi and modulating NMDA receptor levels [145]. CUR (50mg/kg, i.p.) treatment also protected cultured neurons against glutamate induced cytotoxicity by mechanisms required TNFR2 [146]. Interestingly, CUR can act as a PPAR-γ agonist and reduces inflammatory responses by inhibiting NF-κB nuclear translocation and decreasing COX-2 levels in Aβ-treated astrocytes, which results in decrease of IL-1, IL-6, and TNF-α level [147,148].

Another interesting approach was provided by Gomez *et al*. (2007) that designed a CUR -based small molecule catalyst template for accelerating oxidative protein folding in ER through novel non-redox chemistry and by this (10-50 µM), reduced the misfolding/unfolding abnormalities within cell [149].

### 4.6. Huperzine A

HupA is a naturally occurring sesquiterpene alkaloid found in the firmoss *Huperzia serrate *[150]. HupA has been used in China for the treatment of fever and swelling. HupA has been also used as a dietary supplement for improving memory [151].

HupA displays a good pharmacokinetic profile with rapid absorption, wide distribution in body and low to moderate rate of elimination [152]. Encapsulated and micosphered formulations of HupA were designed to control the release of HupA and thereby increase its efficacy [153-157]. HupA is a strong AChEI and acts by a mechanism similar to rivastigmine, donepezil, and galantamine. New tacrine-HupA hybrids (Huprines) are potent AChEI and significantly attenuate Aβ-induced oxidative injury [158-160]. Clinical trials showed minimal adverse cholinergic effects of HupA such as dizziness, nausea, gastroenteric symptoms, headaches, and depressed heart rate. These minimal cholinergic side effects would be an advantage of HupA compared to other AChEIs for the treatment of AD [161].

HupA potentially beneficial actions include inhibiting apoptotic factors, such as caspase-3, Bax and p53, and regulating the expression and secretion of NGF [162]. 

HupA (0.1 mg/kg) improved learning and memory of Tg mice in Morris water maze test, mainly by activating PKC/MAPK pathway, α-secretases, BACE, and increasing phospho GSK-3β [158,163]. HupA (167 and 500 µg/kg, nasal gel) also activated Wnt/β-catenin signaling pathway. HupA neuroprotection was associated with reduced amyloid plaques and oligomeric Aβ level in the cortex and hippocampus [164]. HupA (0.1 and 1 µM) could also antagonize NMDA receptor and potassium current [165].

HupA also affected mitochondrial function by restoring enzymatic activity of respiratory chain complexes and preventing Aβ-induced ATP reduction and mitochondrial swelling [166]. 

### 4.7. Rosmarinic Acid

Rosmarinic acid (RA) is a polyphenol antioxidant carboxylic acid existed in many Lamiaceae herbs used commonly as culinary herbs, such as lemon balm (*Melissa officinalis*), rosemary (*Rosmarinus officinalis*), oregano (*Origanum vulgare*), sage (*Salvia officinalis*), thyme and peppermint [167,168]. RA possesses many biological activities including antioxidant, anti-inflammatory, anticancer, antiviral, antibacterial, and neuroprotective effects [169,170]. No severe side effect has been reported for RA.

Orally administered RA undergoes cleavage of ester bonds, selective para-dehydroxylation, methylation, and sulfate-conjugation. Approximately 83% of the total amount of these metabolites excretes in urine, 8 to 18 h after RA administration to rats [171,172].

RA (0.25-4 mg/kg, i.p.) significantly prevented Aβ-induced memory impairments, mainly by targeting NF-κB and TNF-α [173,174]. RA also reduced tau hyperphosphorylation [175]. RA (1-10 µM) could also inhibit apoptotic pathways by inhibiting ROS formation, DNA fragmentation, and caspase-3 activation [175].

### 4.8. Luteolin

Lu is a yellow crystalline flavonoid widely distributed in plant families of Bryophyta, Pteridophyta, Pinophyta and Magnoliophyta. Dietary sources of Lu include carrots, peppers, celery, olive oil, peppermint, thyme, rosemary and oregano [176]. Lu possesses a variety of pharmacological activities, including antioxidant, anti-inflammatory, antimicrobial, anticancer, and neuroprotective activities [177].

Pharmacokinetic analysis showed that Lu converts to glucuronides during passing through the intestinal mucosa. Some Lu could escape the intestinal conjugation and the hepatic sulfation/methylation and presented in plasma as a free Lu [178].

It has been shown that Lu (20-100 µM) efficiently attenuated zinc-induced tau hyperphosphorylation, not only by its antioxidant activity, but also through the regulation of tau phosphatase/kinase system [179]. Moreover, it down-regulated the expression of APP and lowered the secretion of Aβ [180]. Lu inhibited caspase-dependent apoptosis by reducing intracellular ROS generation, increasing SOD activity and reversing mitochondrial membrane potential dissipation [180]. Lu (10-20 µM) concentration-dependently enhanced neuronal cell survival with efficacy higher than and potency similar to vitamin E, in ROS-insulted primary neurons [181]. Lu also activated Nrf2 pathway and induced ERK1/2 activation in neuronal cells [182].

Lu modulation of long-term potentiation formation was mediated not only directly, but also by protecting synapses from the detrimental effects of chronic cerebral hypoperfusion. This effect of Lu (150-450 mg/kg) on learning and memory may be due to the activation of CREB [183].

### 4.9. Apigenin

Apigenin (AP) is a yellow crystalline nonmutagenic bioflavonoid presented in leafy plants and vegetables such as parsley, artichoke, basil and celery [184]. AP possesses anti-inflammatory, antioxidant, and anticancer properties [184]. There is very little evidence to date to suggest that AP promotes adverse metabolic reactions *in vivo* when consumed as part of a normal diet [184, 185].

AP has low solubility and high intestinal permeability. AP could be well absorbed in the whole intestine by different transport mechanisms but its main absorption site is duodenum [186]. It is also a potent inhibitor of CYP450, an enzyme responsible for the metabolism of many pharmaceutical drugs in the body [187].

AP (10-50 µM) reduced apoptotic cell death induced by thapsigargin and brefeldin A, two representative ER stress inducers, by suppressing ROS accumulation and blocking activation of caspase-12 and -3 and cleavage of PARP. It could also reduce ER stress markers, including CHOP, GRP78 and GRP94, the cleavage of ATF6, the phosphorylation of eIF2α and IRE1α. Suppression of MAPK pathway was also observed in AP-treated cells [188].

### 4.10. Berberine

Berberine (BBR) is a quaternary ammonium salt from the protoberberine, group of isoquinoline alkaloids found in *Berberis aquifolium* (Oregon grape), *Berberis vulgaris* (barberry), *Berberis aristata* (tree turmeric), *Hydrastis canadensis* (goldenseal), *Phellodendron amurense* (Amur cork tree), *Coptis chinensis* (Chinese goldthread), and *Tinospora cordifolia *[189].

Many pharmacological activities have been reported for BBR, including antioxidant activity, AChE and butyrylcholinesterase inhibition, monoamine oxidase inhibition, and cholesterol-lowering activity [190]. Gastrointestinal symptoms such as diarrhea, constipation, flatulence and abdominal pain were the main reported side effects of BBR treatment in human subjects [191, 192]. Under ultraviolet light, BBR (1.8 mM) shows a strong yellow fluorescence [193]. It has been reported that the intestinal absorption of BBR is very low. The *in vivo* studies indicated that the bioavailability of the BBR-loaded microemulsion formulation was significantly greater than that of the BBR tablet suspensions, suggesting the microemulsion as a promising oral drug delivery system for BBR [194].

BBR treatment (100 mg/kg, p.o.) significantly ameliorated learning, as well as long-term spatial memory retention in Tg mice [195]. Significantly decrease at the levels of C-terminal fragments of APP and the hyper-phosphorylation of APP and tau was observed in N2a mouse neuroblastoma [195]. These results were gained by the effect of BBR on Akt/GSK3 signaling pathway that regulates APP processing. BBR (1-20 µM) also attenuated tau hyperphosphorylation and Aβ production in HEK293 cells [196,197]. BBR (20 mg/kg, i.p.) could also promote the survival and differentiation of hippocampal precursor cells and axonal regeneration in injured nerves of the peripheral nervous system [198].

In addition, BBR (50 mg/kg, p.o.) significantly inhibited Aβ-stimulated production of inflammatory factors including IL-6, COX-2 and iNOS in BV2 microglia cells [199]. BBR (1-5 µM) strongly inhibited NF-κB by blocking the PI3K/protein kinase B and MAPK pathways [200].

### 4.11. Chitosan and its Derivatives

Chitosan is a linear polysaccharide composed of randomly distributed β-(1-4)-linked D-glucosamine (deacetylated unit) and N-acetyl-D-glucosamine (acetylated unit) [201]. Chitosan is produced commercially by deacetylation of chitin, which is the structural element in the exoskeleton of crustaceans (such as crabs and shrimp) and cell walls of fungi [202]. Most common chitosan side effects are gastric upsets but it’s not severe. No serious adverse effects were reported in clinical studies of chitosan [203, 204].

Chitosan rapidly clots blood and has recently gained approval in the United States and Europe for use in bandages and other hemostatic agents [205]. It is widely used in pharmaceutical science for targeted drug delivery [206]. Chitosan nanoparticles have been studied for delivering rivastigmine and tacrine to CNS for the treatment of AD [207-210]. More interestingly, a chitosan nanocarrier is designed as a nano-vaccine for delivering Aβ into brain [211].

Recent studies suggested that chitosan itself could protect neurons against H_2_O_2_-induced apoptosis by preventing Aβ formation and blocking intrinsic apoptosis pathway. Chitosan (0.1 and 0.5 % w/v) also upregulated Nrf2 (and its downstreams, HO-1 and γ-GCS) and inhibited NF-κB in NT2 neural cells [212]. Chitooligosaccharides (COS; 50-150 µg/ml) showed the same pattern of protection in PC12 cells by decreasing intracellular ROS and Ca^2+^ levels and suppressing apoptosis. It could also increase protective Hsp70 levels while down-regulated Hsp90 expression. Moreover, COS could inhibit the phosphorylation of different MAPKs, whose aberrant phosphorylation has been implicated in AD [213]. COS (25-1000 µg/ml) also had inhibitory effects on BACE-1 and inhibited Aβ-induced AChE activity [214,215].

Water soluble chitosan (10 µg/ml) also prevented inflammatory responses in Aβ-stimulated human astrocytoma cells by decreasing TNF-α and IL-6 levels, and suppressing iNOS expression [216].

## 5. CONCLUSION

Several natural products are used alone or in combination with other neuroprotective compounds to improve memory and cognition in AD patients. The healing power of culinary herbs and medicinal plants attracted researchers’ attention to study natural products as a potentially valuable resource for drug discovery against AD. This review discussed some natural products with potential neuroprotective properties against Aβ with respect to their mechanism of action. Although neuroprotective compounds derived from natural sources are attractive therapeutic alternatives in the treatment of AD, poor bioavailability and low clinical efficacy are the major problems. Using novel pharmaceutical technologies and medicinal chemistry to prepare novel formulations or design new compounds based on natural templates, opens up new window into using natural therapeutics agents against AD.

## Figures and Tables

**Table 1.** Natural Compounds with Protective Effect Against Aβ

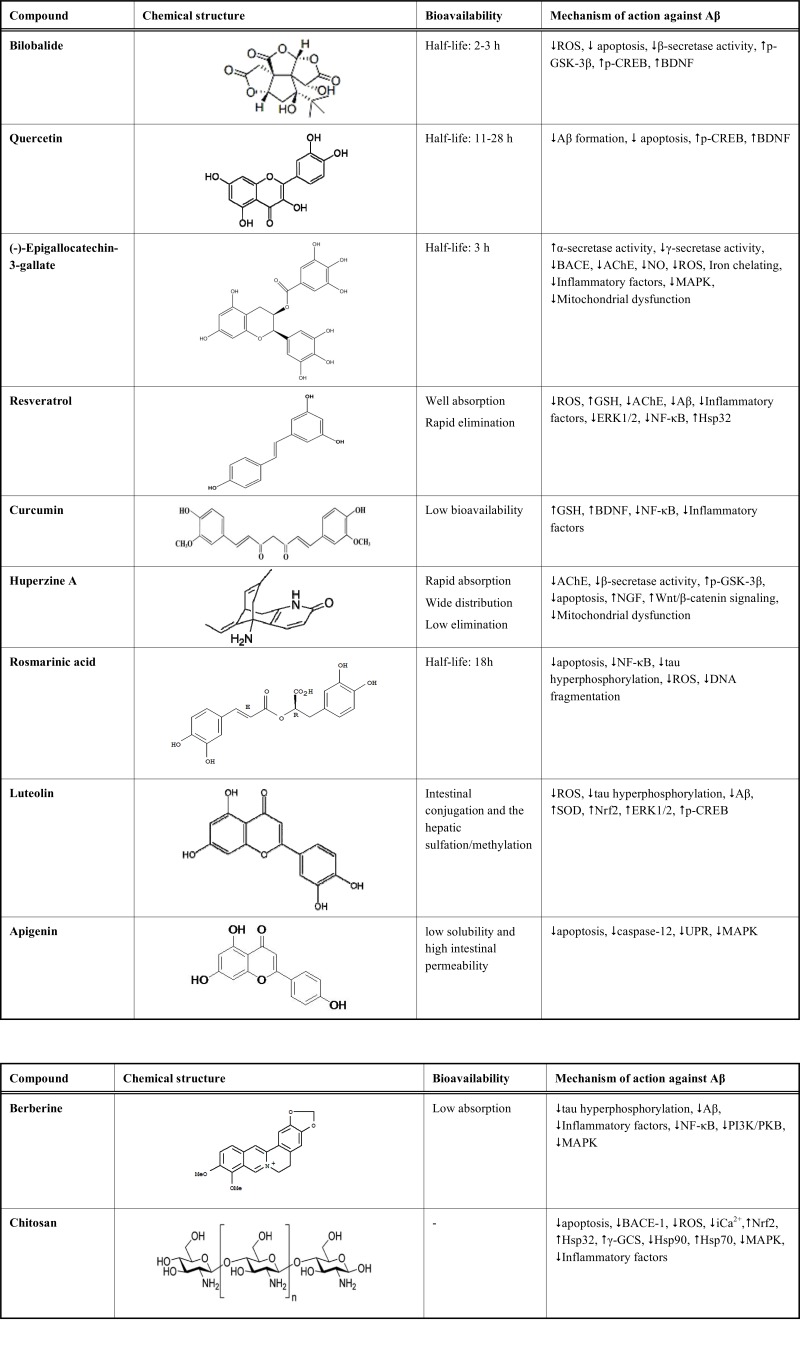

## ABBREVIATIONS

Aβ: = β-amyloid
AChE: = acetyl cholinesterase
AChEI: = acetyl cholinesterase inhibitor
AD: = Alzheimer’s disease
AP: = apigenin
Apaf1: = apoptotic protease activating factor 1
APP: = amyloid precursor protein
ARE: = antioxidant response element
BACE1: = beta-site APP cleaving enzyme 1
BB: = bilobalide
BBB: = blood brain barrier
BBR: = berberine
BDNF: = brain-derived neurotrophic factor
CAT: = catalase
CHOP: = C/EBP homologous protein
COS: = chitooligosaccharides
CREB: = cAMP-response element binding protein
COX: = cyclooxygenase
CUR: = curcumin
eIF2α: = eukaryotic initiation factor 2α
EGCG: = (-)-Epigallocatechin-3-gallate
EOR: = ER overload response
ER: = endoplasmic reticulum
ERAD: = ER-associated protein degradation
γ-GCS: = γ-glutamylcysteine synthetase
GRP: = glucose-regulated protein
GSK-3β: = glycogen synthase kinase 3β
HSP: = heat shock protein
IκB: = inhibitor of κB
IKK: = IκB kinase
IL: = interleukin
IP3: = inositol 1,4,5-triphosphate receptor
IRE1: = inositol-requiring protein 1
Keap1: = Kelch-like ECH-associated protein 1
LPS: = lipopolysaccharide
Lu: = luteolin
MAPK: = mitogen-activated protein kinase
MDA: = malondialdehyde
NF-κB: = nuclear factor-κB
NGF: = nerve growth factor
Nrf2: = NF-E2 related factor 2
NF-κB: = nuclear factor- κB
NMDA: = N-methyl-D-aspartate
NO: = nitric oxide
iNOS: = inducible nitric oxide synthase
PARP: = poly (ADP-ribose) polymerase
PERK: = protein kinase RNA-like ER kinase
PKC: = protein kinase C
PPAR-γ: = peroxisome proliferator-activated receptor γ
PS: = presenilin
QCT: = quercetin
RA: = rosmarinic acid
ROS: = ractive oxygen species
RSV: = Resveratrol
SOD: = superoxide dismutase
SLN: = solid lipid nanoparticles
TNF-α: = tumor necrosis factor
TNFR1: = tumor necrosis factor receptor 1
UPR: = unfolded protein response
